# Successful Reduction of a Backward Dislocation of the Radius and Ulna

**Published:** 1882-07-20

**Authors:** George E. Brewer

**Affiliations:** Buffalo, N. Y.


### THE SUCCESSFUL REDUCTION OF A BACKWARD DISLOCATION OF THE RADIUS AND ULNA SEVEN MONTHS AFTER THE INJURY.

BY GEORGE E. BREWER, M. D., OF BUFFALO, N. Y.

A backward dislocation of the elbow of seven months' standing, reduced by Dr. Julius F. Miner, of Buffalo, recently came under my observation.

The extreme rarity of such cases, and the readiness with which reduction was accomplished, led me to a careful study of the subject as presented by surgical authors.

I was very much surprised to see how little was said in our books, or published in our journals; the whole subject being dismissed with the almost unanimous conclusion that such reductions were impracticable if not impossible.

I was also much surprised to find how uniformly unsuccessful had been the efforts of even the most experienced surgeons in attempting to better this condition, some authors even going so far as to assert that it should not be attempted after the sixth week.

Hamilton, in his valuable work on fractures and dislocations, cites but two instances of its successful reduction after seven months, one in his own practice and one reported by Starch.

With Dr. Miner's permission I report the following case, trusting that it may prove instructive to those interested in the subject of old dislocations.

Edward McCarty, aged twenty-one, of Crawford county, Pennsylvania, in August last, received a fall which resulted in a backward dislocation of the radius and ulna. The injury was such as to baffle all attempts at reduction made by neighboring physicians. March 15th, about seven months after the injury, he consulted Dr. Miner, who found, upon examination, that the dislocation was complete, both bones of the forearm being distinctly felt resting on the humerus about three inches above their normal position, the coracoid process of the ulna firmly imbedded in the olecranon fossa, and the arm immovably extended.

The patient was at once given a private room in the Buffalo General Hospital, when, on the following day, Dr. Miner, in the presence of, and assisted by, many of his professional friends, renewed the attempt at reduction.

The patient was thoroughly etherized. Forcible extension was then made upon the arm. This was followed by an unsuccessful attempt at flexion, clearly showing further extension necessary before flexion could be accomplished. The tendon of the triceps was now divided about an inch above its attachment to the olecranon. Very powerful extension was then made (the combined efforts of four men), this, with forcible flexion, pronation, and supination was continued for nearly three-quarters of an hour. Then measurement, the ease with which the arm could be flexed and absence of deformity, all indicated that the dislocation had been successfully reduced.

The arm was dressed at right angles, without a splint, and the patient placed in bed.

March 17th.—Considerable swelling of the parts. Passive motion attended with pain. Pulse, 110; temperature 99°.

March 19th.—Patient sitting up. Passive motion. Pulse, 90; temperature, 98°.

March 22d.—One week after the operation. Motion without pain, but limited, owing to the œdematous condition of surrounding tissues. Patient left the hospital and went to his home in Pennsylvania, with every indication that complete recovery and good motion would eventually be obtained.

In these old dislocations of the forearm, especially where the displacement is considerable, the contracted triceps offers the chief muscular resistance to replacement.

Dixi Crosby, of New Hampshire, successfully replaced two old luxations of this kind by fracturing the olecranon. This method has also been successfully practiced by Hamilton and others. Subcutaneous section of the tendon of the triceps seems to possess all the merits, while it lacks some of the disadvantages attending the fracture of the olecranon.

Since the above was written, Dr. Miner informs me that in October, 1869, assisted by his friend and colleague, the late Professor Sanford Eastman, M. D., he reduced a similar dislocation in the same manner. Dr. Harrington, who also assisted Dr. Miner at that time, says that the dislocation was of nearly a year's standing. As no record was made of the case at the time, he is unable to state positively just how much time had elapsed since the injury.

Dr. Miner suggests that the reason more cases of the kind have not been reported, is not that such dislocations have not been reduced, but, as in his first case, no records were preserved.—*N. Y. Med. Record.*

